# Non-GM Genome Editing Approaches in Crops

**DOI:** 10.3389/fgeed.2021.817279

**Published:** 2021-12-15

**Authors:** Zheng Gong, Ming Cheng, Jose R. Botella

**Affiliations:** Plant Genetic Engineering Laboratory, School of Agriculture and Food Science, The University of Queensland, Brisbane, QLD, Australia

**Keywords:** transgene-free, genome editing, virus induced genome editing, CRISPR (clustered regularly interspaced short palindromic repeat)/Cas9 (CRISPR associated protein 9)-mediated genome editing, non-GM approach, crops, RNPs

## Abstract

CRISPR/Cas-based genome editing technologies have the potential to fast-track large-scale crop breeding programs. However, the rigid cell wall limits the delivery of CRISPR/Cas components into plant cells, decreasing genome editing efficiency. Established methods, such as *Agrobacterium tumefaciens*-mediated or biolistic transformation have been used to integrate genetic cassettes containing CRISPR components into the plant genome. Although efficient, these methods pose several problems, including 1) The transformation process requires laborious and time-consuming tissue culture and regeneration steps; 2) many crop species and elite varieties are recalcitrant to transformation; 3) The segregation of transgenes in vegetatively propagated or highly heterozygous crops, such as pineapple, is either difficult or impossible; and 4) The production of a genetically modified first generation can lead to public controversy and onerous government regulations. The development of transgene-free genome editing technologies can address many problems associated with transgenic-based approaches. Transgene-free genome editing have been achieved through the delivery of preassembled CRISPR/Cas ribonucleoproteins, although its application is limited. The use of viral vectors for delivery of CRISPR/Cas components has recently emerged as a powerful alternative but it requires further exploration. In this review, we discuss the different strategies, principles, applications, and future directions of transgene-free genome editing methods.

## 1 Introduction

Plant breeding aims to produce improved crop varieties with enhanced agronomic traits and better nutrition qualities for a growing human population. However, traditional breeding methods are often slow, and the production of new traits is restricted by the species’ existing genetic variation pool (Voytas and Gao, 2014; Baltes et al., 2017; Mao et al., 2019; Wang et al., 2019; Nasti and Voytas, 2021).

Genome editing allow plant breeders to manipulate crop genomes at the nucleotide level with high precision. In particular, the advent of prokaryotic-derived Clustered Regularly Interspaced Short Palindromic Repeats (CRISPR)/CRISPR associated protein (Cas) systems and its use in plant genome editing has been a crucial turning point towards a new era of crop breeding. Cas9 and Cas12a, are two popular RNA guided engineered nucleases (RGENs) which mediate genome editing, directed by the sequence-specific pairing of a guide RNA (gRNA) to the target DNA (Jinek et al., 2012). CRISPR/Cas systems have been widely adopted for a variety of applications, including gene disruption by the production of insertion-deletion mutations (indels) (Mao et al., 2013), site-specific sequence integration (Čermák et al., 2015; Wang et al., 2017), transcriptional control (Lowder et al., 2015; Pan et al., 2021), and base editing (Zong et al., 2017) among others (Zhan et al., 2021). Precise genome editing tools like CRISPR/Cas gives plant breeders unprecedented control over the breeding process at the molecular level. Combined with our current knowledge and the rapid progress in plant genomics, the versatile CRISPR/Cas systems can efficiently introduce genetic variations into the plant genome for crop improvement.

The efficient introduction of genome editing reagents into plants remains one of the grand challenges for this technology (Zhang et al., 2018; Mao et al., 2019; Yang, 2020). Indeed, the minimal CRISPR/Cas complex, a large Cas protein (>140 kDa for Cas9 and Cas12a) and gRNA needs to be delivered across the rigid cell wall, into the nucleus of plant cells. Currently, delivery mostly relies on *Agrobacterium*-mediated or biolistic genetic transformation methods (Baltes et al., 2017). However, the stable integration of transgenes in both methods lead to an array of issues, such as the integration of transgenes at random sites in the plant genome which can disrupt essential genes or result in variable transgene expression (Sun et al., 2016; van Kregten et al., 2016; Liu et al., 2019). In addition, many plant species and elite crop varieties are recalcitrant to genetic transformation and/or plant regeneration (Sun et al., 2016). Removal of transgenes through segregation is also difficult or even impossible in asexually propagated or highly heterozygous crops. Most importantly, many jurisdictions impose heavy regulations on Genetically Modified Organisms (GMOs) which restrict their development, commercialization and use in agriculture (Turnbull et al., 2021). The general public’s perception of GMO plants is likewise negative, leading to a shift away from GMO products.

CRISPR/Cas genome editing tools enable precise and traceable modifications that are no different from naturally occurring genetic variations selected during conventional breeding (Voytas and Gao, 2014; Pacher and Puchta, 2017; Zhang et al., 2018). Many countries such as the USA, Japan and Australia exclude some or all kinds of genome-edited crops from GMO regulation if they are free of transgenes or foreign DNA (Pacher and Puchta, 2017; Tsuda et al., 2019; Entine et al., 2021). Even the European Union, which regulates all gene-edited plants as GMOs, released a study that recognized its regulations as “not fit for purpose for some new genomics techniques” (European Commission, 2021). Thus, plant genome editing approaches that avoid transgenesis have recently gained considerable attention. Protocols using Cas9 ribonucleoproteins or transient gene expression with viral vectors have emerged as promising tools for genome editing, whilst avoiding foreign DNA integration. These methods do not involve GM and are collectively named as transgene-free genome editing. Here, we will briefly discuss major advances in transgene-free plant genome editing.

## 2 Ribonucleoproteins

**FIGURE 1:**
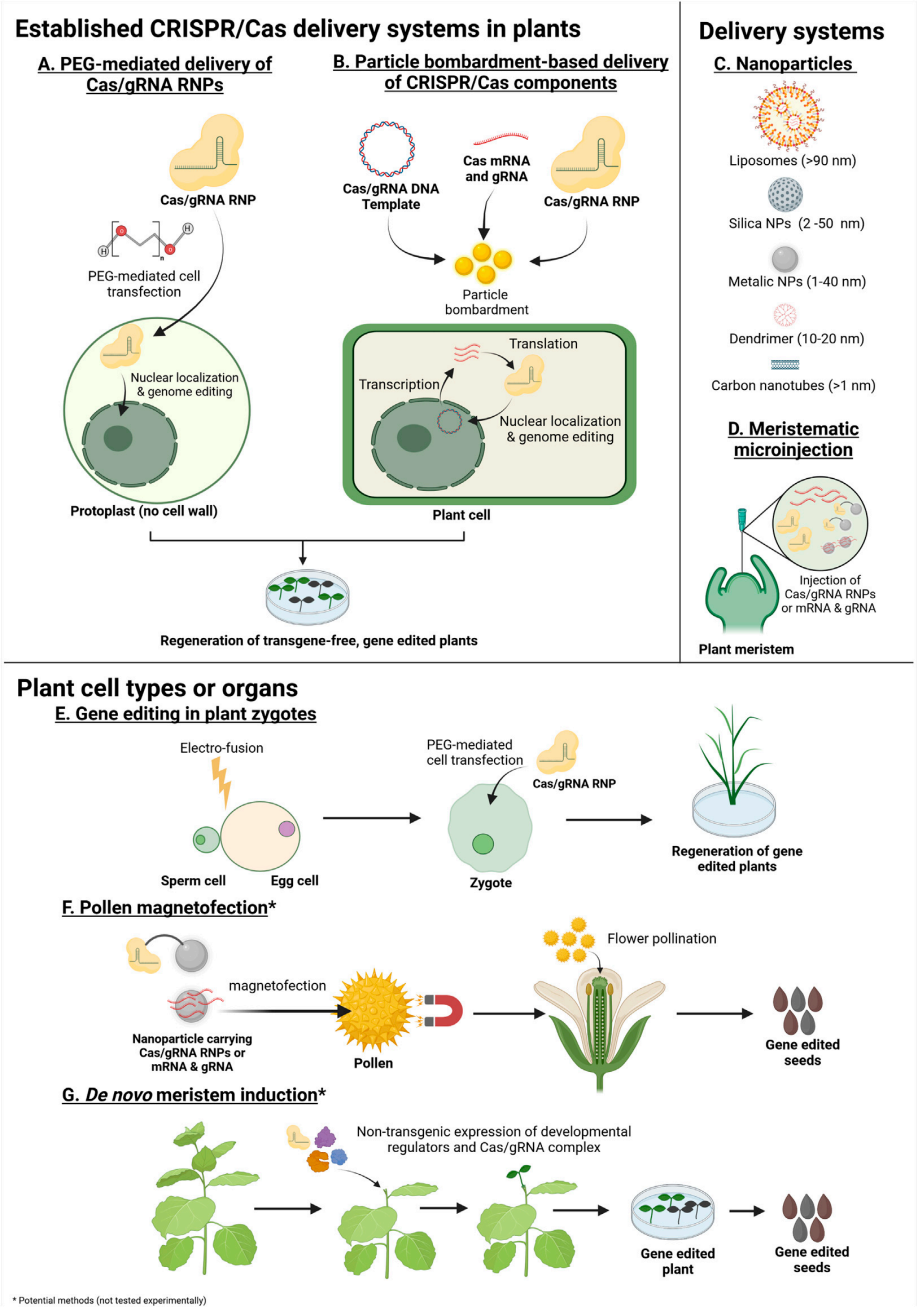
Methods with potential for transgene-free genome editing in plants using *in vitro*/chemically synthesized Cas mRNA and gRNA, DNA templates or Cas/gRNA ribonucleoproteins (RNPs) (Created with BioRender.com). The asterisk (*) indicates methods which have not been experimentally tested. **(A,B)** Established, GM-free systems for the delivery of CRISPR/Cas/gRNA into plants. **(A)** Polyethylene glycol (PEG)-mediated delivery of preassembled Cas/gRNA RNPs into plant protoplasts. PEG mediates the uptake of Cas/gRNA RNPs into protoplast cells. The Cas/gRNA complex enters the nucleus and induces genome editing. The protoplasts are then regenerated to produce transgene-free gene edited plants. **(B)** Particle bombardment-based delivery of preassembled Cas/gRNA RNPs, Cas mRNA plus gRNA or Cas/gRNA DNA expression cassettes. The CRISPR components are loaded onto particles and introduced into plant cells using a gene gun or biolistic device. The Cas/gRNA complexes are localized to the nucleus and induce genome editing. Regeneration from bombarded plant tissue without selection produces gene edited plants whilst avoiding transgenesis (in a large proportion of the regenerated plants). **(C,D)** Potential delivery systems for the application of transgene-free genome editing in plants. **(C)** Common types of nanoparticles currently used in biotechnology. Nanoparticles, such as carbon nanotubes, have been explored as delivery systems for DNA/RNA and protein into mesophyll through stomata pores (around 10 nm). Other nanoparticles, larger than 10 nm, can be introduced into plants by chemical, or physical methods. Therefore, nanoparticles could act as a carrier for the delivery of genome editing reagents into plant cells. Note that nanoparticles are not restricted to spherical forms. **(D)** Plant meristematic microinjection using phytoinjectors. Phytoinjectors could potentially be adopted for the injection of Cas mRNA and gRNA, RNPs or nanoparticle-bound genome editing reagents. **(E–G)** Potential target cell types and organs for transgene-free genome editing. **(E)** Gene editing in plant zygotes. The isolated sperm and egg cells are electro-fused to form zygotes. Early zygotes lack cell walls and the Cas/gRNA RNPs are transfected with PEG. Zygotes are then regenerated to obtain gene edited plants. **(F)** Gene editing using pollen. Nanoparticles carrying Cas mRNA and gRNAs or Cas/gRNA RNPs could be transfected into pollen through magnetofection. The transfected pollen is used to pollinate a flower to produce transgene-free gene edited seeds. **(G)** Non-transgenic delivery/transient expression of developmental regulators and CRISPR/Cas reagents in plant organs may produce gene edited, *de novo* meristems which can be cultured to obtain gene edited seeds.

The direct use of CRISPR/Cas ribonucleoproteins (RNPs) is the most obvious approach to achieve transgene-free genome editing. RNPs can be easily assembled by combining purified Cas protein and *in vitro* transcribed or chemically synthesized gRNA before being introduced into cells using chemical or physical delivery methods (Figure 1). As such, the use of RNPs circumvents the design and multi-step construction of recombinant vectors, as well as promoter and codon optimisation issues. Most importantly, the CRISPR/Cas RNPs are only transiently present in plant cells prior to degradation by proteases and nucleases (Woo et al., 2015; Zhang et al., 2016; Liang et al., 2017; Banakar et al., 2019). This significantly reduces mosaicism and off-target effects caused by extended exposure of genomic DNA to CRISPR reagents in the conventional DNA delivery systems (Woo et al., 2015; Zhang et al., 2016). The use of RNPs completely avoids transgenesis and should not fall under regulatory oversight (Wolter and Puchta, 2017). Furthermore, RNPs mediate genome editing shortly after cell transfection, as gene transcription and translation is not necessary, enabling the development of approaches for rapid evaluation of multiple CRISPR/Cas systems efficiency in plant tissues (Banakar et al., 2020; Kim et al., 2020).

### 2.1 Protoplast Transformation

The use of CRISPR/Cas9 RNPs was first reported in 2014, for human cell mutagenesis (Kim et al., 2014), and have since been extensively adopted for plant genome editing in a variety of plant species including *Arabidopsis thaliana*, rice, lettuce, tobacco (Woo et al., 2015; Kim et al., 2017), petunia (Subburaj et al., 2016; Yu et al., 2021), grapevine, apple (Malnoy et al., 2016), maize (Svitashev et al., 2016), wheat (Liang et al., 2017; Liang et al., 2018), soybean (Kim et al., 2017), potato (Andersson et al., 2018; González et al., 2020; Nicolia et al., 2021b), cabbage (Murovec et al., 2018; Park et al., 2019; Lee et al., 2020), banana (Wu et al., 2020), pepper (Kim et al., 2020), witloof (De Bruyn et al., 2020), carrot (Klimek-Chodacka et al., 2021), and tomato (Nicolia et al., 2021a). In most cases, polyethylene glycol-calcium (PEG-Ca^2+^)-mediated cell transfection was the method used to deliver the RNPs into plant protoplasts. However, PEG-mediated transformation may cause cell cytotoxicity leading to limited reproducibility. Surprisingly, Andersson et al. (2018) reported that a large proportion of the regenerated plants contained inserts at the target site, containing either random fragments of potato chromosomal DNA or originating from the DNA template used to synthesize the gRNA. Despite the success of PEG-mediated delivery of RNPs in certain transformation-recalcitrant species, few plant species have been satisfactorily regenerated from protoplasts (Yue et al., 2021). Moreover, genome instability caused by protoplast regeneration is not infrequent (Fossi et al., 2019). Due to a lack of well-established and species-specific protoplast isolation and regeneration techniques, especially for monocotyledonous plants, the adoption of PEG-mediated RNP genome editing has been limited thus far (Yue et al., 2021). Other useful strategies for the delivery of genes or proteins to mammalian cells, such as electroporation and lipofection, have also been tested in plants. Electroporation of *Chlamydomonas Reinhardtii* cells with CRISPR/Cas9 RNPs resulted in approximately 1% editing efficiency (Baek et al., 2016). Electro-transfection of CRISPR/Cas9 RNPs into cabbage protoplasts provided a 1.6% increase in editing efficiency compared to PEG-mediated transfection (Lee et al., 2020). Lipofection was demonstrated to transport RNPs into negatively charged tobacco BY2 protoplasts by mixing the CRISPR/Cas9 RNPs with positively charged cationic lipids, resulting in a 6% editing efficiency (Liu et al., 2020).

### 2.2 Particle Bombardment

Particle bombardment can be used to deliver CRISPR/Cas RNPs into multiple tissues such as immature embryos, leaf discs and calli and is not limited by plant-host range (Altpeter et al., 2005). Major cereal crops, such as rice (Banakar et al., 2020), wheat (Liang et al., 2017; Liang et al., 2019), *Brassica* (Murovec et al., 2018) and maize (Svitashev et al., 2016) have been successfully edited by bombardment with 0.6 µm gold particles coated with CRISPR/Cas RNPs using a helium gene gun. Mutated plants were generated from bombarded embryogenic wheat calli in 6–8 weeks without selection (Liang et al., 2017). In general, the mutagenesis efficiency using particle bombardment of CRISPR/Cas RNPs is modest or low, requiring large-scale mutant screening (Banakar et al., 2019). The addition of a selectable marker plasmid increases the editing efficiency of CRISPR/Cas RNPs, but this approach can result in DNA integration into the plant genome (Svitashev et al., 2016; Banakar et al., 2019). Biolistic bombardment may also result in genome damage, which could lead to phenotypic changes or reduced fitness (Liu et al., 2019).

### 2.3 Future Directions

#### 2.3.1 Zygotes and Pollen as Delivery Targets

Other plant material, such as zygotes and pollen have the potential to avoid protoplast regeneration. Rice zygotes are created by uniting isolated egg and sperm cells, a process known as gamete fusion. Cell walls are immature during the early stages of gamete fusion, allowing Toda et al. (2019) to perform PEG-mediated transfection of preassembled CRISPR/Cas9 RNPs. After 30–40 days of culture, 14–64% of the generated plants from the zygotes contained CRISPR-induced mutations (Toda et al., 2019). This approach is promising and could be applied to other species with available gamete fusion and regeneration protocols. Pollen manipulation could also circumvent many of the tissue culture and regeneration problems. Pollen grains in many plant species are permeable through apertures of 5–10 µm in diameter and thus are theoretically amenable to the delivery of preassembled RNPs using nanotechnological approaches and are discussed in the next section (Zhao et al., 2017).

#### 2.3.2 Nanoparticles for Cargo Delivery

Nanoparticles (<100 nm) have been successfully used to deliver DNA, RNA and proteins into plant cells (Martin-Ortigosa et al., 2013; Zhao et al., 2017; Demirer et al., 2019; Demirer et al., 2020). Polyethyleneimine (PEI)-coated Fe_3_O_4_ magnetic nanoparticles were used to carry exogenous DNA plasmids into the pollen grains of several dicot plants, including cotton, pepper, pumpkin and cocozelle (Zhao et al., 2017). The DNA-loaded nanoparticles were combined with pollen in solution and subjected to a magnetic field to enhance the movement of the particles to the bottom of the recipient and into the pollen grains in a process known as magnetofection (Zhao et al., 2017). Artificial pollination using magnetofected pollen produced genetically modified seeds (Zhao et al., 2017). This approach could theoretically be used to introduce preassembled RNPs into pollen instead of DNA but unfortunately attempts to use magnetofection by several research groups have failed, casting some doubts about the efficiency of the method (Vejlupkova et al., 2020).

In mammalian cells, nanoparticle delivery of CRISPR/Cas9 RNPs have been accomplished (Lee et al., 2017), however no nanomaterial-mediated transgene-free CRISPR/Cas genome editing has been reported in plants so far (Ranjan et al., 2017; Sanzari et al., 2019; Demirer et al., 2021). The use of conjugated nanomaterials/RNPs as delivery method and subsequent release methods by enzymatic or light-mediated cleavage is an attractive possibility for future research (Ahmar et al., 2021; Demirer et al., 2021; Nadakuduti and Enciso-Rodríguez, 2021; Wang et al., 2021).

## 3 Virus Induced Genome Editing

**FIGURE 2:**
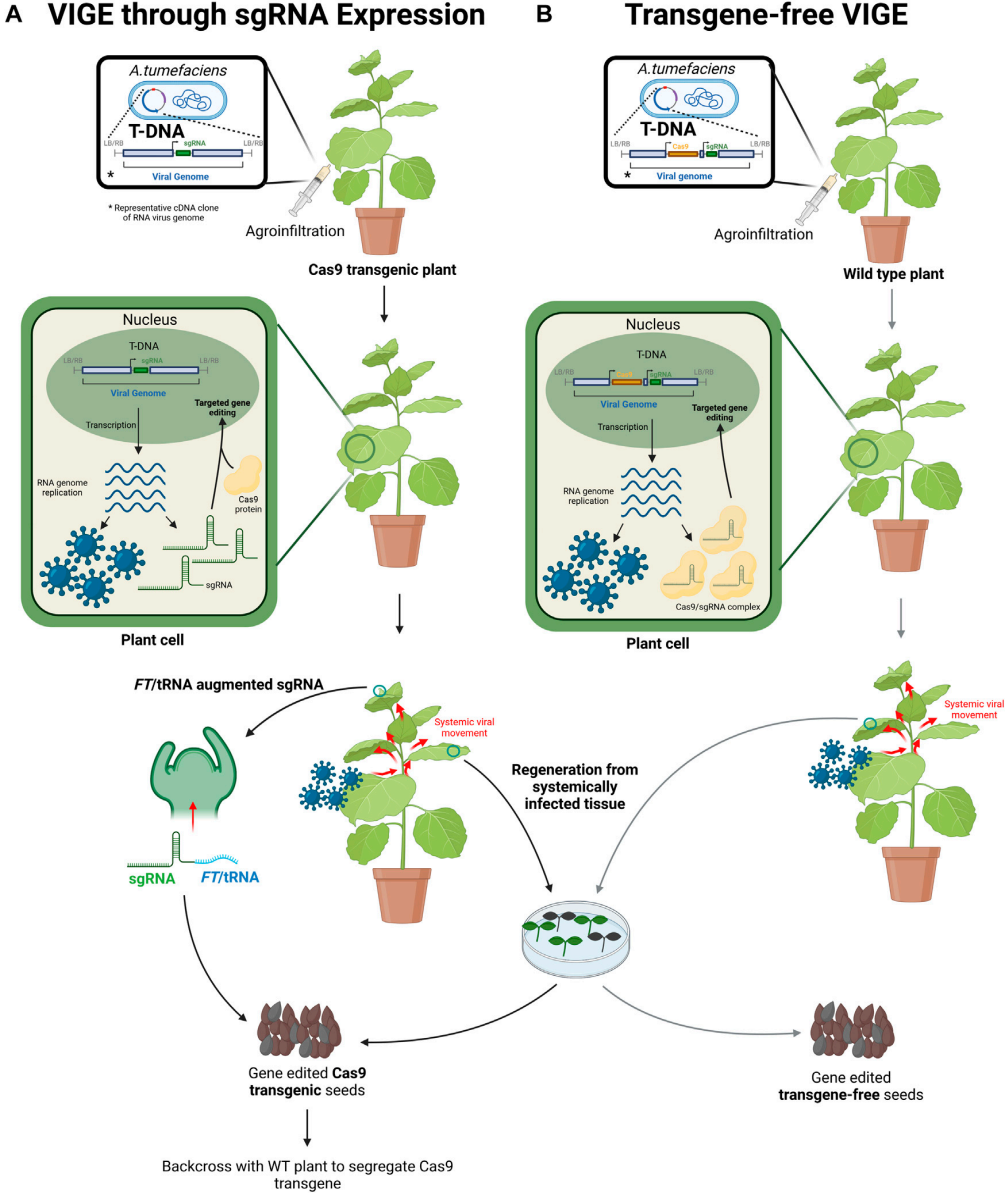
Virus induced genome editing (VIGE) systems in plants (Created with BioRender.com). **(A)** VIGE through gRNA expression. The gRNA is cloned into the complementary DNA (cDNA) of an RNA virus genome in a binary vector. *A. tumefaciens* carrying the binary vector with the recombinant viral cDNA is agroinfiltrated into a leaf on Cas9 transgenic plants. The viral cDNA is expressed to produce viral RNA which self-replicates. gRNAs transcribed from the viral vector are bound by the Cas9 protein expressed from the plant integrated transgene. The Cas9/gRNA complex is localized in the nucleus where it induces targeted gene editing. Viral genomes are encapsidated into recombinant virions which exit the plant cell inducing systemic infection. When gRNAs are fused to *FT* or tRNAs, the augmented gRNA may travel and enter meristematic cells. Gene editing in meristematic cells can produce edited Cas9 transgenic seeds. Alternatively, systemically infected leaf tissue can be used for plant regeneration. Genome edited Cas9 transgenic seeds are obtained from regenerated plants. The gene edited transgenic Cas9 progenies can be backcrossed with wild type (WT) plants to segregate the Cas9 transgene. **(B)** Transgene-free VIGE system. Certain viruses are capable of carrying and expressing cassettes containing Cas9 and gRNAs. The Cas9 gene and gRNA are introduced into the viral genome cDNA in a binary vector and agroinfiltrated into WT plants. The viral cDNA is expressed to produce viral RNA which self-replicates. Cas9 protein and gRNA are transiently expressed from the viral genome to form a complex. The Cas9/gRNA complex is localized to the nucleus for targeted genome editing. Viral genomes are encapsidated into recombinant virions exiting the plant cell and induces systemic infection. Systemically infected leaf tissue can be used for plant regeneration. The regenerated plants produce transgene-free gene-edited seeds.

Viral vectors are an efficient tool for gene expression in plants (Scholthof et al., 1996) and have been extensively used for foreign and endogenous gene expression as well as targeted gene silencing (Brisson et al., 1984; French et al., 1986; Chapman et al., 1992; Lu, 2003; Giritch et al., 2006; Gao et al., 2013; Torti et al., 2021). The use of viruses offers multiple advantages including 1) Transient and systemic gene expression without the need for transgenesis (Ellison et al., 2021); 2) high gene expression levels (Pogue and Holzberg, 2012); and 3) the availability of a broad range of viruses that can be engineered for gene expression infecting different plant species (Supplementary Table S1 and Supplementary Table S2). Thus, viruses provide an attractive platform for transgene-free delivery of genome editing reagents, providing a promising solution to the delivery bottleneck. In this section, we explore the recent advances in virus induced genome editing (VIGE) (Figure 2). Strategies to insert foreign genes, such as genome editing tools, into viral genomes are well established for many plant viruses (Scholthof et al., 1996; Mortimer et al., 2015) (Supplementary Table S2). The gene-of-interest is inserted into viral genomes, sometimes replacing non-essential viral genes (Chapman et al., 1992). Infection with recombinant viral genomes into plant tissues is predominantly achieved through agroinfiltration (Marillonnet et al., 2004; Marillonnet et al., 2005). Alternatively, infection can be accomplished by mechanical inoculation of the viral genome or the use of previously infected tissue (Takamatsu et al., 1987). Once inside a cell, the viral genome undergoes its replication lifecycle, inducing local gene expression in the inoculated area before producing complete virions and colonising the plant (Lico et al., 2008; Mortimer et al., 2015). Concurrently, the inserted gene-of-interest is expressed systemically alongside the viral infection. Transgene-free expression of foreign genes, such as genome editing reagents, can be achieved if genome integration is not involved in the viral lifecycle.

### 3.1 Genome Editing Using Positive-Strand RNA Viruses

RNA viruses have a strong potential for transgene-free genome editing as they multiply through RNA replication and are not usually reverse transcribed into DNA throughout their lifecycle (Ellison et al., 2021).

#### 3.1.1 Delivery of Zinc Finger Nucleases and Meganucleases

Tobacco rattle virus (TRV) is a bipartite positive-strand RNA virus (PSV) infecting many dicotyledonous plant species (MacFarlane, 2008; Marton et al., 2010; Ali et al., 2015a). TRV has been modified to express a zinc finger nuclease (ZFN), targeting a loss-of-function *GUS* transgene in *Nicotiana benthamiana* and *Petunia hybrida* transgenic lines (Marton et al., 2010). Zinc finger nuclease-mediated editing of *GUS* restored GUS activity producing a visible phenotype upon staining in systemic leaves. In addition, TRV infected tissues from these experiments were used for regeneration. Virus-free and zinc finger nuclease-free seedlings containing edits in the *GUS* gene were identified from T0 seeds of the regenerated plants (Marton et al., 2010). TRV was also used to express a meganuclease targeting *DIHYDROFLAVONOL 4-REDUCTASE* (*DFR*) in *Nicotiana alata* (Honig et al., 2015). DFR is involved in the synthesis of anthocyanins, simplifying the visual identification of mutations by the presence of reduced pigmentation in the *N. alata* purple petals. Analysis of the progeny of three infected plants revealed the presence of two plants containing mutations in one of the two DFR genes present in the genome (Honig et al., 2015).

#### 3.1.2 Delivery of CRISPR/Cas gRNAs

In contrast with zinc finger nucleases and meganucleases, the delivery of CRISPR/Cas reagents with PSVs is notably more difficult. The size of the Cas9 coding region (>4 kb), significantly larger than ZFNs (∼1, 2 kb), create problems for delivery using PSVs due to their limited cargo size. Large insertions also cause genome instability from selective pressure towards viral particles lacking the insert (Walker et al., 2015; Kujur et al., 2021; Tsanova et al., 2021). Initial studies overcame the size problem by using the PSV system to express small gRNAs into transgenic plant lines constitutively expressing Cas9 as a proof-of-concept. PSVs such as TRV, Beet necrotic yellow vein virus (BNYVV), Potato virus X (PVX) and the legume-infecting, Pea early browning virus (PEBV) have been used to express gRNAs in Cas9-positive *N. benthamiana* lines (Ali et al., 2015a; Ali et al., 2015b; Ali et al., 2018; Jiang et al., 2019; Uranga et al., 2021a). Efficient VIGE was detected in systemically infected leaves with the four PSVs, ranging from ∼30 to ∼85% editing efficiency. Furthermore, when PVX infected tissues were used for regeneration of plants by tissue culture, edited seedlings were recovered (Uranga et al., 2021a). It is to note that gRNA delivery and highly efficient gene editing in Cas9 transgenic *N. benthamiana* was also achieved with a DNA virus, Cabbage leaf curl virus (Yin et al., 2015).

For monocotyledonous plants, the tripartite PSV, barley stripe mosaic virus (BSMV) was used to express gRNAs targeting endogenous genes in the agriculturally important crops, wheat and maize using a similar approach to the ones mentioned above (Hu et al., 2019). BSMV showed very high editing efficiency in systemic leaves of Cas9 transgenic wheat (∼62–78%) and maize (∼48%) lines. Expression of gRNAs using the foxtail mosaic virus (FoMV) mediated efficient VIGE in Cas9 transgenic *Setaria viridis* (∼60% in systemic leaves) but efficiency significantly dropped in Cas9 transgenic maize (∼3–6%) (Mei et al., 2019). In both VIGE systems, wheat, maize and *S. viridis* plants were easily infected by rub-inoculation with *N. benthamiana* leaves from plants previously infected by agroinfiltration (Hu et al., 2019; Mei et al., 2019). The possibility of efficient *trans*-species rub-inoculation is especially important for monocots where the introduction of viral genomes into mature plants is difficult, demonstrating the versatility of viral vectors.

Despite the high editing efficiency demonstrated by the above-mentioned PSV vectors in systemic tissues, an important consideration is whether this approach produces gene edits in the progeny of infected plants. Unfortunately, these studies either 1) failed to obtain gene edits in the progeny (FoMV infected *S. viridis*), 2) obtained an extremely low frequency (2/1,320 seedlings from TRV infected *N. benthamiana*) or 3) heritability was not determined (BNYVV, PEBV and BSMV infected *N. benthamiana*, FoMV infected maize).

#### 3.1.3 Heritable Genome Editing Through gRNA Augmentation

To optimize heritable VIGE in CRISPR/Cas systems, Ellison et al. (2020) fused the *A. thaliana FLOWERING LOCUS T* (*FT*) mRNA to the 3’ end of the gRNA in an approach called gRNA augmentation. Substantial evidence suggests that the *FT* mRNA moves systemically in the plant and enters the meristem to induce flowering (Li et al., 2009; Li et al., 2011). The idea behind gRNA augmentation is that the addition of *FT* or other mobility sequences such as tRNAs to the gRNA will confer systemic mobility and access to meristematic cells to produce heritable editing. When transgenic *N. benthamiana* plants overexpressing Cas9 were infected with TRV vectors containing *FT* augmented gRNAs, up to 65% of the progeny contained CRISPR-generated mutations (Ellison et al., 2020). Since the initial report, gRNA *FT* augmentation has been used with other dicotyledon viruses such as PVX and a DNA virus, Cotton leaf crumple virus, generating heritable edits of 22% in *N. benthamiana* and >4% in *A. thaliana* seedlings, respectively (Lei et al., 2021; Uranga et al., 2021a). This strategy has also been implemented in monocot crops such as wheat with surprising results. Cas9 transgenic wheat lines were infected with BSMV expressing either unaugmented gRNAs or augmented gRNAs with wheat *FT* (Li et al., 2021). Unexpectedly, gene edits were present in almost all M1 progeny from plants infected with unaugmented gRNA whereas, the progeny of plants infected with BSMV-*FT* augmented gRNA were rarely edited. To quickly remove the Cas9 transgene, the authors used anthers from plants infected with BSMV-gRNA for pollination of wild type plants. The progeny was self-pollinated to obtain edited plants lacking the Cas9 transgene (Li et al., 2021). This BSMV VIGE system has the potential to circumvent the need for tissue culture in genome editing pipelines involving monocots such as maize and barley which BSMV infects.

#### 3.1.4 Transgene-Free Genome Editing Using Positive-Strand RNA Virus

Despite achieving high gene editing efficiency and sometimes heritable editing, the above discussed approaches are not truly transgene-free as they need to use a Cas9 expressing line as starting plant material for infection. PVX is a monopartite PSV with a filamentous flexible architecture which may allow to incorporate the large Cas9 gene into its RNA genome (Ariga et al., 2020). Recently, PVX was used to transiently express Cas9 and gRNA in *N. benthamiana.* No systemic editing was discussed in this work, suggesting that none was found possibly because the incorporation of the large Cas9 cassette led to defective viral movement. Nevertheless, regeneration of plants from tissues agroinfiltrated with the viral cDNA yielded >50% plants containing mutations with 18% also containing a T-DNA integration. The same strategy was attempted using a nickase Cas9-base editor fusion with >60% of regenerated plants containing base edits while ∼30% contained T-DNA integration (Ariga et al., 2020). The progeny from regenerated plants retained genomic edits but were free of PVX RNA. To avoid T-DNA integration, the authors performed agroinfiltration to establish infection in a source plant and used mechanical inoculation from the source plant into recipient plants. In this way, the recipient plants were never in contact with *Agrobacterium,* eliminating the risk of T-DNA incorporation, thus providing a DNA free method for editing. Unfortunately, this approach proved much less efficient than the direct agroinfiltration with only 2–4% of regenerated pants containing mutations. A non-systemic PSV expression vector based on the tobacco mosaic virus was also developed which expressed both Cas9 and gRNA for gene editing in the presence of *p19*, a viral suppressor of RNA silencing. However, no attempt was made to regenerate gene edited plants (Chiong et al., 2021).

Foxtail mosaic virus (FoMV), from the same *Potexvirus* genus as PVX, has been used to mediate systemic gene editing in a transgene-free fashion (Zhang et al., 2020). *N. benthamiana* leaves were simultaneously agroinfiltrated with a FoMV vector containing a Cas9 expression cassette and a second FoMV vector containing a gRNA cassette targeting the *PHYTOENE DESATURASE* (*PDS*) gene. Sequencing of the targeted genomic region detected no edits but addition of a cassette containing the viral RNA silencing suppressor, *p19,* to the gRNA vector produced gene editing in systemic tissues (Zhang et al., 2020). These results are in line with recent research indicating that RNA silencing suppressors can increase genome editing efficiency (Mao et al., 2018; Zhang et al., 2020; Chiong et al., 2021; Mao et al., 2021). Unfortunately, the authors did not attempt to regenerate plants from systemically infected tissue or test heritable editing in the progeny of infected plants and previous studies have failed to obtain edited seeds (Zhang et al., 2020).

Similarly, Uranga et al. (2021b) achieved transgene-free genome editing using two compatible viruses that can co-infect the same cells. The PSV, Tobacco etch virus (TEV) was used to express CRISPR/Cas12a by replacing the *NIb* gene in the TEV genome. Another PVX virus expressing both the gRNA and the *NIb* gene to supplement the recombinant TEV was constructed. Both recombinant viral genomes were co-agroinfiltrated into wild type *N. benthamiana*, mediating around 20% gene editing efficiency in systemic leaves. Although the authors did not investigate the heritability of gene editing, we believe that regeneration from systemically infected tissue is likely to produce gene-edited progenies (Uranga et al., 2021b).

### 3.2 Genome Editing Using Negative-Strand Viruses

Rhabdoviruses are a group of negative-strand RNA viruses containing a large genome (>10 kb) (Jackson et al., 2005). Rhabdoviruses have large cargo capacities and high gene stability, making them a suitable candidate for transgene-free genome editing (Walker et al., 2015; Dietzgen et al., 2017). The Barley yellow striate mosaic virus (BYSMV) was the first monocot-infecting rhabdovirus developed into an expression system (Gao et al., 2019). When a BYSMV-based vector was used to express Cas9 and gRNA in *N. benthamiana* plants, Sanger sequencing successfully detected different indels in the infiltrated area, but systemic gene editing was not discussed. The authors also explored the use of BYSMV as a gene expression platform for planthoppers and monocots such as barley, wheat and *Setaria italica*; however, genome editing was not investigated in these systems (Gao et al., 2019).

The *Sonchus* yellow net virus (SYNV), a rhabdovirus infecting dicotyledonous species, has been also used to express Cas9 and gRNA in *N. benthamiana*. SYNV-mediated gene editing generated mutations in systemic tissues with high efficiency for a *GFP* transgene (77–91%), as well as three endogenous genes (40–79% for *PDS*, 53%–91% for *RDR6*, and 79–91% for *SGS3*) (Ma et al., 2020). Up to 93% of plants regenerated from systemically infected leaves contained some form of gene editing which also produced edited seeds in the next generation. Unfortunately, heritable editing was not detected in seeds from the initially infected plants (Ma et al., 2020). Although the host range of SYNV is extremely limited, there are multiple rhabdoviruses infecting a diverse range of plant species, thus, a suite of rhabdovirus expression platforms can be developed for different plant species.

### 3.3 Future Directions

Viruses have the potential to become an efficient and versatile vector for the delivery of CRISPR genome editing reagents, however, several important limitations still need to be addressed, the most important being the large size of Cas9. Although some of the available methods can achieve seed heritability using mobility sequences, they still require the use of transgenic Cas9 lines while transgene-free genome editing using viral vectors involves tissue culture for plant regeneration (Figure 2) (Ellison et al., 2020; Ma et al., 2020; Zhang et al., 2020). It will be interesting to investigate whether heritable editing can be achieved by combining mobility sequences in a rhabdovirus, FoMV or TEV-PVX based VIGE platform (Uranga et al., 2021b). Several recent studies have also discovered new RGENs with dramatically reduced size compared to Cas9 (∼1,000–1,400 amino acids). For example, the phage derived CasΦ is a compact RGEN (∼700–800 amino acids) capable of generating gene edits in *A. thaliana* protoplasts, albeit at low efficiency (Pausch et al., 2020). Obligated mobile element-guided activity (OMEGA) is another class of transposon-encoded RGENs with a reduced size (∼400 amino acids) (Altae-Tran et al., 2021). Miniature CRISPR associated RGENs such as Cas12f are also being explored for gene editing capability and engineered for improved efficiency (Bigelyte et al., 2021; Xu et al., 2021). The discovery and optimization of these smaller genome editing tools may facilitate delivery with germline infecting PSVs such as TRV or BSMV.

## 4 Discussion

Transgene-free genome editing can be an ideal technology to breed sustainable and more nutritious crops; however, several plant-specific challenges need to be overcome before it can achieve its full potential. One of the most significant hurdles is the delivery of CRISPR/Cas components into plant cells, whilst avoiding transgenesis. The use of preassembled CRISPR/Cas RNPs is arguably the most direct option, but the available methods utilise specific plant cells or organs such as protoplasts, immature embryos, or zygotes followed by regeneration of whole plants. These processes are technically difficult, inefficient, and are not available for many economically important crops. Viral vectors have also emerged as promising tools for transgene-free genome editing, especially because they can be used in full plants and there is a large number of virus vectors for gene silencing which are available to be implemented for VIGE (Supplementary Table S2). Multiple VIGE systems have been developed for transient and systemic expression of Cas9 and/or gRNAs with efficient editing in dicotyledonous and monocotyledonous species (Supplementary Table S1). But efficient heritable mutations were only achieved through plant regeneration. Some viral systems and innovative approaches such as gRNA augmentation with mobility sequences can achieve heritable gene editing through seeds but they still need to use transgenic Cas9 plants. The ideal VIGE system should combine transgene-free and heritability at acceptable efficiency to alleviate the genome editing delivery bottleneck. The use of nanomaterials as delivery systems for plants is only starting but we expect to see rapid advances with this approach. Alternative strategies such as the use of mRNA-based genome editing (Zhang et al., 2016) or the *de novo* induction of meristems (Maher et al., 2020) have been reported but have not yet being been adopted by the wider research community. Even though steady and continuous progress is being made, the field is in need of completely new approaches and the ideal solution might involve development of new and disruptive technologies.
