# Supplementary material for: Non-GM Genome Editing Approaches in Crops
Source: Front Genome Ed. 2021 Dec 15;3:817279. doi: 10.3389/fgeed.2021.817279 (PMC8715957; doi:10.3389/fgeed.2021.817279)
Supplement: Supplementary file 1 [file Table1.DOCX]

| Table S1. Examples of RNA virus vectors used for virus induced gene editing (VIGE) | | | | | | | |
| --- | --- | --- | --- | --- | --- | --- | --- |
| Virus Name | **Genus** | **RNA genome** | **Plant of application** | **Host range** | **Delivery of genome editing components** | **Heritable editing through seeds?** | **Reference** |
| BNYVV: Beet necrotic yellow vein virus | Benyvirus | ssRNA (+) | *N. benthamiana* | *N. benthamiana,* *Chenopodium quinoa* and spinach | sgRNA only | Not tested | (Jiang et al., 2019) |
| BSMV: Barley stripe mosaic virus | Hordeivirus | ssRNA (+) | *N. benthamiana,* wheat, maize | Barley, maize, oats, wheat | sgRNA only | Yes, in wheat | (Hu et al., 2019; Li et al., 2021) |
| BYSMV: Barley yellow striate mosaic virus | Cytorhabdovirus | ssRNA (-) | *N. benthamiana* | *N. benthamiana,* *Sertaria italica* | Cas9 + sgRNA | Not tested | (Gao et al., 2019) |
| FoMV: Foxtail mosaic virus | Potexvirus | ssRNA (+) | *N. benthamiana*; *Setaria viridis*, maize | Multiple monocotyledons | sgRNA only | No edited seeds in *S.viridis*; not tested in maize | (Mei et al., 2019) |
|  |  |  | *N. benthamiana* |  | Cas9 + sgRNA | Not tested | (Zhang et al., 2020) |
| PEBV: Pea early browning virus | Tobravirus | ssRNA (+) | *N. benthamiana* | Legumes | sgRNA only | Not tested | (Ali et al., 2018) |
| PVX: Potato virus X | Potexvirus | ssRNA (+) | *N. benthamiana* | Multiple dicotyledons | sgRNA only | Yes, using *FT* augmented sgRNA | (Uranga et al., 2021) |
|  |  |  |  |  | Cas9 + sgRNA |  | (Ariga et al., 2020) |
| SYNV: Sonchus yellow necrotic virus | Nucleorhabdovirus | ssRNA (-) | *N. benthamiana* | Species from *Solanaceae* family | Cas9 + sgRNA | No edited seeds | (Ma et al., 2020) |
| TMV: Tobacco mosaic virus | Tobamovirus | ssRNA (+); expression vector | *N. benthamiana* | Crops, weeds, and ornamentals | sgRNA only; Cas9 + sgRNA | Not tested | (Chiong et al., 2021) |
| TRV: Tobacco rattle virus | Tobravirus | ssRNA (+) | *A. thaliana* | Over 400 host species | sgRNA only | Not tested | (Ali et al., 2018) |
|  |  |  | *N. benthamiana* |  | Zinc finger nuclease | Not tested | (Marton et al., 2010) |
|  |  |  | *N. alata* |  | Meganuclease | Yes | (Honig et al., 2015) |
|  |  |  | *N. benthamiana* |  | sgRNA only | Yes, very low efficiency with unaugmented sgRNA; high efficiency with sgRNA augmented with *FT*/tRNA | (Ali et al., 2015a; Ali et al., 2015b) |
