# Supplementary material for: Non-GM Genome Editing Approaches in Crops
Source: Front Genome Ed. 2021 Dec 15;3:817279. doi: 10.3389/fgeed.2021.817279 (PMC8715957; doi:10.3389/fgeed.2021.817279)
Supplement: Supplementary file 2 [file Table2.DOCX]

| **Table S2. RNA virus vectors developed for virus induced gene silencing (VIGS) with potential for VIGE applications** | | | | | |
| --- | --- | --- | --- | --- | --- |
| **Virus Name** | **Genus** | **RNA genome** | **Plant of application** | **Host range** | **Reference** |
| ALSV: Apple latent spherical virus | Cheravirus | ssRNA(+) | *N. benthamiana, A. thaliana,* cucurbits, legumes, tomato, pepper, strawberry | N. benthamiana, fruit trees, vegetables, jegumes and ornamental flowers | (Igarashi et al., 2009; Kasajima et al., 2017; Li et al., 2019a; Li et al., 2019b) |
| Bamboo mosaic virus with its associated satellite RNA | Potexvirus | ssRNA(+) | *N. benthamiana,* *Brachypodium distachyon* | Bamboo, *N. benthamiana, Brachypodium distachyon, Gomphrena globosa*, *Chenopodium amaranticolor* | (Liou et al., 2014) |
| BMV: Brome mosaic virus | Bromovirus | ssRNA(+) | Barley, wheat and maize | Poaceae family including Barley, maize, rice and other monocotyledons | (Ding et al., 2006; Pacak et al., 2010) |
| BPMV: Bean pod mottle virus | Comovirus | ssRNA(+) | Glycine max | Legume | (Zhang and Ghabrial, 2006) |
| BSMV: Barley stripe mosaic virus | Hordeivirus | ssRNA(+) | *N. benthamiana,* barley, rice, wheat and maize | Barley, maize, oats, wheat | (Holzberg et al., 2002; Lacomme et al., 2003; Scofield et al., 2005; Jarugula et al., 2018) |
| CGMMV: Cucumber green mottle mosaic virus | Tobamovirus | ssRNA(+) | *N. benthamiana* & Cucurbits (watermelon, melon, cucumber and bottle gourd) | Bitter gourd, bottle gourd, cucumber, melon, pumpkin, ridged gourd, smooth loofah gourd, snake gourd watermelon, West Indian gherkin, white gourd, zucchini, and weeds. | (Dombrovsky et al., 2017; Liu et al., 2020) |
| CMV: Cucumber mosaic virus | Cucumovirus | ssRNA(+) | *N. benthamiana*, chilli peppers, legume species, maize, soybean, tomato | Many monocotyledons and dicotyledons | (Ogawa et al., 2015; Wang et al., 2016) |
| CTV: *Citrus tristeze virus* | Closterovirus | ssRNA(+) | Citrus | Citrus | (Killiny, 2020) |
| CymMV: Cymbidium mosaic virus | Potyviruses | ssRNA(+) | *Phalaenopsis equestris* | Orchidaceae family | (Lu et al., 2007) |
| FoMV: Foxtail mosaic virus | Potexvirus | ssRNA(+) | Green foxtail, maize, sorghum, sweet corn | Multiple monocotyledons | (Mei et al., 2016) |
| PEBV: pea early browning virus | Tobravirus | ssRNA(+) | *N. benthamiana,* *Pisum sativum* | Legumes | (Constantin et al., 2004; Grønlund et al., 2008) |
| PVX: Potato virus X | Potexvirus | ssRNA(+) | *N. benthamiana,* potato, tomato, tobacco | Multiple dicotyledons | (Bilgin et al., 2003; Lu, 2003; Faivre-Rampant et al., 2004; Lin et al., 2008; Wang et al., 2014) |
| TRSV: Tobacco ringspot virus | Nepovirus | ssRNA (+) | *N. benthamiana,* *A. thaliana*, melon, oriental melon, cucumber, Glycine max | Herbaceous and woody plants (soybeans, cucurbits...etc.) | (Zhao et al., 2016) |
| TRV: Tobacco rattle virus | Tobravirus | ssRNA(+) | Apple, citrus, cotton, *Cysticapnos vesicaria*, eggplant, grape berry, kiwifruit, litchi, loquat, lycium, mulberry, peach, pear, pepper, pitaya, sea buckthorn, strawberry, sweet sherry, tomato, *Xanthoceras sorbifolium* | Over 400 host species | (Pang et al., 2013; Liu et al., 2021) |
| TMV: Tobacco mosaic virus | Tobamovirus | ssRNA(+) | *N. benthamiana* | Wide including tobacco and Solanaceae family | (Kumagai et al., 1995) |
| TYMV: Turnip yellow mosaic virus | Tymovirus | ssRNA(+) | *A. thaliana, Brassica rapa* | Brassicaceae family | (Pflieger et al., 2008; Yu et al., 2018) |

**Reference for Supplementary Table 1:**

Bilgin, D.D., Liu, Y., Schiff, M., and Dinesh-Kumar, S.P. (2003). P58IPK, a Plant Ortholog of Double-Stranded RNA-Dependent Protein Kinase PKR Inhibitor, Functions in Viral Pathogenesis. *Developmental Cell* 4(5)**,** 651-661. doi: 10.1016/s1534-5807(03)00125-4.

Constantin, G.D., Krath, B.N., Macfarlane, S.A., Nicolaisen, M., Elisabeth Johansen, I., and Lund, O.S. (2004). Virus-induced gene silencing as a tool for functional genomics in a legume species. *The Plant Journal* 40(4)**,** 622-631. doi: 10.1111/j.1365-313x.2004.02233.x.

Ding, X.S., Schneider, W.L., Chaluvadi, S.R., Mian, M.A.R., and Nelson, R.S. (2006). Characterization of a Brome mosaic virus Strain and Its Use as a Vector for Gene Silencing in Monocotyledonous Hosts. *Molecular Plant-Microbe Interactions®* 19(11)**,** 1229-1239. doi: 10.1094/mpmi-19-1229.

Dombrovsky, A., Tran-Nguyen, L.T.T., and Jones, R.A.C. (2017). Cucumber green mottle mosaic virus: Rapidly Increasing Global Distribution, Etiology, Epidemiology, and Management. *Annual Review of Phytopathology* 55(1)**,** 231-256. doi: 10.1146/annurev-phyto-080516-035349.

Faivre-Rampant, O., Gilroy, E.M., Hrubikova, K., Hein, I., Millam, S., Loake, G.J., et al. (2004). Potato Virus X-Induced Gene Silencing in Leaves and Tubers of Potato. *Plant Physiology* 134(4)**,** 1308-1316. doi: 10.1104/pp.103.037507.

Grønlund, M., Constantin, G., Piednoir, E., Kovacev, J., Johansen, I.E., and Lund, O.S. (2008). Virus-induced gene silencing in Medicago truncatula and Lathyrus odorata. *Virus Research* 135(2)**,** 345-349. doi: 10.1016/j.virusres.2008.04.005.

Holzberg, S., Brosio, P., Gross, C., and Pogue, G.P. (2002). Barley stripe mosaic virus-induced gene silencing in a monocot plant. *The Plant Journal* 30(3)**,** 315-327. doi: 10.1046/j.1365-313x.2002.01291.x.

Igarashi, A., Yamagata, K., Sugai, T., Takahashi, Y., Sugawara, E., Tamura, A., et al. (2009). Apple latent spherical virus vectors for reliable and effective virus-induced gene silencing among a broad range of plants including tobacco, tomato, Arabidopsis thaliana, cucurbits, and legumes. *Virology* 386(2)**,** 407-416. doi: 10.1016/j.virol.2009.01.039.

Jarugula, S., Willie, K., and Stewart, L.R. (2018). Barley stripe mosaic virus (BSMV) as a virus-induced gene silencing vector in maize seedlings. *Virus Genes* 54(4)**,** 616-620. doi: 10.1007/s11262-018-1569-9.

Kasajima, I., Ito, M., Yamagishi, N., and Yoshikawa, N. (2017). "Apple Latent Spherical Virus (ALSV) Vector as a Tool for Reverse Genetic Studies and Non-transgenic Breeding of a Variety of Crops." Springer International Publishing), 513-536.

Killiny, N. (2020). The efficacy of Citrus tristeza virus as a vector for virus induced gene silencing in Huanglongbing-affected citrus. *Tropical Plant Pathology* 45(3)**,** 327-333. doi: 10.1007/s40858-020-00357-6.

Kumagai, M.H., Donson, J., Della-Cioppa, G., Harvey, D., Hanley, K., and Grill, L.K. (1995). Cytoplasmic inhibition of carotenoid biosynthesis with virus-derived RNA. *Proceedings of the National Academy of Sciences* 92(5)**,** 1679-1683. doi: 10.1073/pnas.92.5.1679.

Lacomme, C., Hrubikova, K., and Hein, I. (2003). Enhancement of virus-induced gene silencing through viral-based production of inverted-repeats. *Plant J* 34(4)**,** 543-553. doi: 10.1046/j.1365-313x.2003.01733.x.

Li, C., Hirano, H., Kasajima, I., Yamagishi, N., and Yoshikawa, N. (2019a). Virus-induced gene silencing in chili pepper by apple latent spherical virus vector. *J Virol Methods* 273**,** 113711. doi: 10.1016/j.jviromet.2019.113711.

Li, C., Yamagishi, N., Kasajima, I., and Yoshikawa, N. (2019b). Virus-induced gene silencing and virus-induced flowering in strawberry (Fragaria × ananassa) using apple latent spherical virus vectors. *Horticulture Research* 6(1). doi: 10.1038/s41438-018-0106-2.

Lin, Z., Hong, Y., Yin, M., Li, C., Zhang, K., and Grierson, D. (2008). A tomato HD-Zip homeobox protein, LeHB-1, plays an important role in floral organogenesis and ripening. *The Plant Journal* 55(2)**,** 301-310. doi: 10.1111/j.1365-313x.2008.03505.x.

Liou, M.R., Huang, Y.W., Hu, C.C., Lin, N.S., and Hsu, Y.H. (2014). A dual gene‐silencing vector system for monocot and dicot plants. *Plant Biotechnology Journal* 12(3)**,** 330-343. doi: 10.1111/pbi.12140.

Liu, G., Li, H., and Fu, D. (2021). Applications of virus-induced gene silencing for identification of gene function in fruit. *Food Quality and Safety* 5. doi: 10.1093/fqsafe/fyab018.

Liu, M., Liang, Z., Aranda, M.A., Hong, N., Liu, L., Kang, B., et al. (2020). A cucumber green mottle mosaic virus vector for virus-induced gene silencing in cucurbit plants. *Plant Methods* 16(1). doi: 10.1186/s13007-020-0560-3.

Lu, H.-C., Chen, H.-H., Tsai, W.-C., Chen, W.-H., Su, H.-J., Chang, D.C.-N., et al. (2007). Strategies for Functional Validation of Genes Involved in Reproductive Stages of Orchids. *Plant Physiology* 143(2)**,** 558-569. doi: 10.1104/pp.106.092742.

Lu, R. (2003). High throughput virus-induced gene silencing implicates heat shock protein 90 in plant disease resistance. *The EMBO Journal* 22(21)**,** 5690-5699. doi: 10.1093/emboj/cdg546.

Mei, Y., Zhang, C., Kernodle, B.M., Hill, J.H., and Whitham, S.A. (2016). A Foxtail mosaic virus Vector for Virus-Induced Gene Silencing in Maize. *Plant Physiology***,** pp.00172.02016. doi: 10.1104/pp.16.00172.

Ogawa, K., Murota, K., Shimura, H., Furuya, M., Togawa, Y., Matsumura, T., et al. (2015). Evidence of capsaicin synthase activity of the Pun1-encoded protein and its role as a determinant of capsaicinoid accumulation in pepper. *BMC Plant Biology* 15(1). doi: 10.1186/s12870-015-0476-7.

Pacak, A., Strozycki, P.M., Barciszewska-Pacak, M., Alejska, M., Lacomme, C., Jarmołowski, A., et al. (2010). The brome mosaic virus-based recombination vector triggers a limited gene silencing response depending on the orientation of the inserted sequence. *Archives of Virology* 155(2)**,** 169-179. doi: 10.1007/s00705-009-0556-9.

Pang, J., Zhu, Y., Li, Q., Liu, J., Tian, Y., Liu, Y., et al. (2013). Development of Agrobacterium-Mediated Virus-Induced Gene Silencing and Performance Evaluation of Four Marker Genes in Gossypium barbadense. *PLoS ONE* 8(9)**,** e73211. doi: 10.1371/journal.pone.0073211.

Pflieger, S.P., Blanchet, S., Camborde, L., Drugeon, G.L., Rousseau, A., Noizet, M., et al. (2008). Efficient virus-induced gene silencing in Arabidopsis using a âone-stepâ TYMV-derived vector. *The Plant Journal* 56(4)**,** 678-690. doi: 10.1111/j.1365-313x.2008.03620.x.

Scofield, S.R., Huang, L., Brandt, A.S., and Gill, B.S. (2005). Development of a Virus-Induced Gene-Silencing System for Hexaploid Wheat and Its Use in Functional Analysis of the Lr21-Mediated Leaf Rust Resistance Pathway. *Plant Physiology* 138(4)**,** 2165-2173. doi: 10.1104/pp.105.061861.

Wang, R., Yang, X., Wang, N., Liu, X., Nelson, R.S., Li, W., et al. (2016). An efficient virus-induced gene silencing vector for maize functional genomics research. *The Plant Journal* 86(1)**,** 102-115. doi: 10.1111/tpj.13142.

Wang, Y., Cong, Q.-Q., Lan, Y.-F., Geng, C., Li, X.-D., Liang, Y.-C., et al. (2014). Development of new potato virus X-based vectors for gene over-expression and gene silencing assay. *Virus Research* 191**,** 62-69. doi: 10.1016/j.virusres.2014.07.018.

Yu, J., Yang, X.D., Wang, Q., Gao, L.W., Yang, Y., Xiao, D., et al. (2018). Efficient virus-induced gene silencing in Brassica rapa using a turnip yellow mosaic virus vector. *Biologia plantarum* 62(4)**,** 826-834. doi: 10.1007/s10535-018-0803-6.

Zhang, C., and Ghabrial, S.A. (2006). Development of Bean pod mottle virus-based vectors for stable protein expression and sequence-specific virus-induced gene silencing in soybean. *Virology* 344(2)**,** 401-411. doi: 10.1016/j.virol.2005.08.046.

Zhao, F., Lim, S., Igori, D., Yoo, R.H., Kwon, S.-Y., and Moon, J.S. (2016). Development of tobacco ringspot virus-based vectors for foreign gene expression and virus-induced gene silencing in a variety of plants. *Virology* 492**,** 166-178. doi: <https://doi.org/10.1016/j.virol.2016.02.025>.
